# Conditional cell reprogramming involves non-canonical β-catenin activation and mTOR-mediated inactivation of Akt

**DOI:** 10.1371/journal.pone.0180897

**Published:** 2017-07-10

**Authors:** Frank A. Suprynowicz, Christopher M. Kamonjoh, Ewa Krawczyk, Seema Agarwal, Anton Wellstein, Fadeke A. Agboke, Sujata Choudhury, Xuefeng Liu, Richard Schlegel

**Affiliations:** Center for Cell Reprogramming, Georgetown University School of Medicine, Washington, DC, United States of America; Univerzitet u Beogradu, SERBIA

## Abstract

The combination of irradiated fibroblast feeder cells and Rho kinase inhibitor, Y-267362, converts primary epithelial cells growing *in vitro* into an undifferentiated adult stem cell-like state that is characterized by long-term proliferation. This cell culture method also maintains the proliferation of adult epithelial stem cells from various tissues. Both primary and adult stem cells retain their tissue-specific differentiation potential upon removal of the culture conditions. Due to the ability to modulate the proliferation and differentiation of the cells, this method is referred to as conditional reprogramming and it is increasingly being used in studies of tumor heterogeneity, personalized medicine and regenerative medicine. However, little is known about the biology of these conditionally reprogrammed (CR) cells. Previously we showed that β-catenin activation, a hallmark of stem cells *in vivo*, occurs in CR human ectocervical cells (HECs). Here we show that β-catenin-dependent transcription is necessary for the induction of epithelial stem cell markers, and that β-catenin is activated via a non-canonical pathway that is independent of Wnt and Akt/GSK-3. Active Akt actually decreases due to increased mTOR signaling, with a consequent increase in dephosphorylated, active GSK-3. Despite the increase in active GSK-3, β-catenin associates with protein phosphatase 2A (PP2A) and is activated. Inhibition of PP2A catalytic activity reduces both the level of active β-catenin and the acute induction of stem cell markers, suggesting an important role for PP2A in the activation of β-catenin. Moreover, we demonstrate similar results using human prostate and breast cells, indicating that these changes are not restricted to ectocervical epithelial cells and may represent a more fundamental property of conditional reprogramming.

## Introduction

Primary epithelial cells co-cultured with gamma-irradiated fibroblast feeder cells and a Rho kinase (ROCK) inhibitor, Y-267362, can be propagated indefinitely *in vitro* without the use of exogenous gene expression [1,2]. These conditions conditionally reprogram the epithelial cells to an undifferentiated adult stem cell-like state that maintains tissue-specific lineage commitment [3], such that the cells differentiate normally upon removal of the reprogramming conditions [1–3]. Therefore, conditionally reprogrammed (CR) cells offer possibilities for regenerative medicine without the genetic instability, tumorigenicity and altered antigenicity of embryonic stem cells (ESCs) and induced pluripotent stem cells (iPSCs) [4–6]. In addition, karyotype-stable normal and tumor cells from the same patient can be propagated using the CR technique [2], providing opportunities for genetic characterization and *in vitro* chemotherapeutic testing [7].

The activation (stabilization, accumulation and nuclear translocation) of β-catenin is essential to maintain epithelial stem cell populations *in vivo* [8], and levels of nuclear β-catenin rapidly increase during conditional reprogramming of primary human ectocervical cells (HECs) *in vitro* [3]. In the nucleus, β-catenin associates with lymphocyte enhancer factor/T cell factor (LEF/TCF) family transcription factors to activate genes that control fundamental aspects of growth, differentiation and cell division [9]. In the present study, we show that β-catenin-dependent transcription is required for the induction of epithelial stem cell markers in CR HECs.

β-catenin is destabilized (targeted for ubiquitination and proteosomal degradation) by glycogen synthase kinase-3 (GSK-3)-mediated phosphorylation at three N-terminal residues (S33, S37 and T41), and is stabilized by protein phosphatase 2A (PP2A)-mediated dephosphorylation of these sites [10]. While PP2A binds directly to β-catenin [11], GSK-3 and β-catenin must both be tethered to the axin scaffolding protein in order for phosphorylation to occur [10]. Typically, β-catenin is activated either as a result of GSK-3 inactivation (via its phosphorylation by AKT) or by the recruitment of axin, β-catenin and GSK-3 to a ternary complex of Frizzled, phosphorylated Dishevelled and phosphorylated LRP5/6 at the plasma membrane (Wnt signaling) [10,12]. Here we show that neither of these canonical signaling pathways activates β-catenin in CR cells. Akt activity decreases during conditional reprogramming and, consequently, the level of dephosphorylated (active) GSK-3 increases. LRP6 phosphorylation does not increase in CR cells and LGK-974, a potent inhibitor of Wnt secretion, does not prevent the activation of β-catenin. Rather, β-catenin is dephosphorylated and activated as a result of increased association with PP2A.

## Materials and methods

### Cell culture

Primary HECs were cultured from discarded and de-identified cervical tissue after hysterectomy for benign uterine disease and passaged 1–2 times in keratinocyte growth medium (KGM; Life Technologies, Carlsbad, CA) without feeder cells before freezing [13]. HECs from at least 12 different patient tissue samples were used in this study. The Georgetown University Institutional Review Board granted an exemption to allow use of cervical tissue because the identity of patients from which the discarded tissue was obtained was not known. PrECs and HECs are commercially available and were purchased from Lonza (Walkersville, MD). PrECs were maintained in KGM and HMECs were maintained in mammary epithelial growth medium (MEGM; Lonza).

Primary epithelial cells were cultured in KGM (or MEGM) for 1–2 passages to acclimate to the synthetic media before experiments. The cells were then plated in KGM/MEGM or, at the same time, plated on 75% confluent gamma-irradiated 3T3 J2 murine fibroblasts (feeder cells) in F-medium containing 10 μM Y-27632 (Enzo Life Sciences, Farmingdale, NY) as previously described [3]. All cultures were processed for immunoblotting or RNA isolation 2–3 d later. (KGM/MEGM cells always were processed 3 d later). To inhibit β-catenin-dependent transcription, cultures were treated with 25 μM iCRT3 (25 mM stock solution dissolved in DMSO; EMD/Millipore, Billerica, MA). Stock solutions of LGK-974 (Selleckchem, Houston, TX) and okadaic acid (Enzo Life Sciences, Farmingdale, NY) were dissolved in DMSO at concentrations of 20 mM and 1 mM respectively.

### Western blotting

J2 feeder cells were removed from 10-cm tissue co-culture dishes by treatment with 0.02% EDTA (in PBS) for 5 min at 37°C. The epithelial cells were then washed with PBS containing 1 mM Na_3_VO_4_ (Sigma, St. Louis, MO) and 1 μM okadaic acid and scraped into 0.5 ml RIPA buffer containing protease inhibitors [14], 1 mM Na_3_VO_4_ and 1 μM okadaic acid at 4°C. Lysates were mixed with 0.5 ml 2X SDS-PAGE sample buffer (4% SDS, 20% glycerol, 0.01% bromophenol blue, 125 mM Tris-HCl, pH 6.8) and subjected to ultrasonic disruption for 10 s twice at room temperature. After heating at 110°C for 10 min, aliquots were removed for determination of protein concentrations using the Bio-Rad DC protein assay (Bio-Rad, Hercules, CA). Mercaptoethanol (5%) was added to the remaining portion of the samples, which were incubated for 15 min at 37°C and frozen. SDS polyacrylamide mini-gels (Novex, Winston-Salem, NC) were transferred to Protran 0.45 μm pore nitrocellulose membranes (Whatman, Shrewsbury, MA) and labeled using antibodies listed in Table 1 according to the manufacturer’s instructions. Alkaline phosphatase-conjugated secondary antibodies and CDP-Star chemiluminescence detection reagent were purchased from Applied Biosystems (Foster City, CA). BioMax MR film (Carestream Health; Rochester, NY) was used to visualize immunoblots, which subsequently were digitally imaged using a Canon (Melville, NY) CanoScan 8800F scanner. Densitometry was performed using Kodak MI software (Rochester, NY). This software allows the user to draw a rectangle around a band and then duplicate the rectangle to encircle the band in all other lanes, even if a signal is not visible by eye in the region where a band would be located. It then determines the total pixel density inside each rectangle. The density inside a rectangle located well away from all lanes is also determined and is subtracted from the other values as background. Each time point is expressed relative to the level of GAPDH at the same time point, with KGM values set to 1.0. Results typical of two or more experiments are shown. P-values were calculated using the chi square method and are significant at p < .01.

**Table 1:** Antibodies used in this study.

Antigen	Source	Antibody type	Application	Dilution
Integrin α6	Abcam 124924	R monoclonal	WB	1:2000
Integrin β1	Abcam 134179	R monoclonal	WB	1:500
p63α	Santa Cruz 8344	R polyclonal	WB	1:1000
CD44	Cell Signaling 5640	M monoclonal	WB	1:1000
β-catenin	Cell Signaling 2698	M monoclonal	WB	1:1000
β-catenin	Cell Signaling 2677	M monoclonal	IF	1:300
GAPDH	Cell Signaling 2118	R monoclonal	WB	1:1000
Non-phospho-(active) β-catenin (S33, S37, T42)	Cell Signaling 8814	R monoclonal	WB	1:1000
p-β-catenin (S45)	Cell Signaling 9564	R polyclonal	WB	1:1000
p-GSK-3β (S9)	Cell Signaling 9322	R monoclonal	WB	1:1000
GSK-3β	Cell Signaling 9315	R monoclonal	WB; IP	1:1000; 1:50
GSK-3β	Cell Signaling 9832	M monoclonal	WB; IP	1:1000; 1:50
PR55α	Cell Signaling 2290	R monoclonal	WB; IP	1:1000; 1:25
PR55α	Cell Signaling 5689	M monoclonal	WB	1:1000
PP2A C subunit	Cell Signaling 2038	R polyclonal	WB	1:1000
PP2A C subunit	Abcam 66597	M monoclonal	IP	1:75
p-mTOR (S2448)	Cell Signaling 5536	R monoclonal	WB	1:1000
p-p70 S6K (T389)	Cell Signaling 9205	R polyclonal	WB	1:1000
p-Rictor (T1135)	Cell Signaling 3806	R monoclonal	WB	1:1000
p-Akt (S473)	Cell Signaling 4060	R monoclonal	WB	1:2000
p-Akt (T308)	Cell Signaling 4056	R monoclonal	WB	1:1000
Akt	Cell Signaling 4691	R monoclonal	WB	1:1000
p-PDK1 (S241)	Cell Signaling 3438	R monoclonal	WB	1:1000
Lamins A+ C	Cell Signaling 4777	M monoclonal	WB	1:2000
p-GSK-3α (S21)	Cell Signaling 9316	R monoclonal	WB	1:1000
GSK-3α	Cell Signaling 4337	R monoclonal	WB	1:1000
p-β-catenin (S675)	Cell Signaling 4176	R monoclonal	WB	1:1000
p-LRP6 (S1490)	Cell Signaling 2568	R ployclonal	WB	1:1000
LRP6	Cell Signaling 3395	R monoclonal	WB	1:1000
E-cadherin	Abcam 40772	R monoclonal	IF	1:100

R: rabbit; M: mouse

For peptide blocking LRP6 and phospho-LRP6 immunoblots, a 10-fold excess (1.4 μg/ml) of phospho-LRP6 (S1490) peptide corresponding to the LRP6 phospho-epitope (Abcam # ab192756; Cambridge, MA) was incubated with the primary antibody for 30 m at room temperature before labeling immumoblot membranes overnight at 4°C.

Uncropped images of all Western blots are shown as supplementary figures (S1–S8 Figs).

### Immunoprecipitation

J2 feeder cells were removed from 10-cm tissue culture dishes by EDTA treatment. Epithelial cells were scraped into 1.2 ml RIPA buffer containing protease inhibitors, Na_3_VO_4_ and okadaic acid and were further disrupted by vortex mixing for 10 min at 4°C. Up to 4 μg of antibody (see Table 1) and 40 μl of 50% (v/v) Thermo/Peirce (Waltham, MA) ImmunoPure Plus protein A or protein G agarose bead suspension was added to the lysates, followed by end-over-end rotation for 90 min at 4°C. The beads were then washed 3 times with RIPA buffer, 3 times with PBS, and bound proteins eluted in 2X SDS-PAGE sample buffer (6 min at 110°C).

### Cell fractionation

Nuclear and non-nuclear fractions were prepared from epithelial cells (after removing J2 cells with EDTA) using the Thermo/Pierce NE-PER extraction kit according to the manufacturer’s instructions.

### Immunofluorescence microscopy

HECs were grown for 3 days on 22 mm square glass coverslips in KGM, or on 75% confluent irradiated J2 fibroblasts in F-medium containing 10 μM Y-27632. The J2 cells were not removed prior to fixation, but could easily be distinguished from colonies of HECs, and are not present in any micrographs shown in this paper. Procedures for paraformaldehyde fixation, saponin permeabilization, immunolabeling and DNA counterstaining have been described in detail [15]. To visualize nuclear β-catenin, coverslips were washed with TBS (150 mM NaCl, 10 mM Tris-HCl, pH 7.4) and extracted at 4°C for 20 m with TBS containing 1 mM MgCl_2_, 1% (w/v) Triton X-100 (TX-100), 1 mg/ml aprotinin, leupeptin and pepstatin, and 0.5 mM PMSF prior to fixation. The antibodies used for immunolabeling are listed in Table 1. A Zeiss Axioskop microscope (Carl Zeiss, Dublin, CA) equipped with 63X oil immersion objective lens, Orca-ER charge-coupled device camera (Hamamatsu, Bridgewater, NJ) and Openlab digital imaging software (Perkin-Elmer, Waltham, MA) was used for microphotography.

### Real-time quantitative RT-PCR

qRT-PCR was performed to determine levels of Axin2, CD44 and c-myc mRNA (relative to endogenous GAPDH mRNA) as described previously [16], using the following primers:

Axin2 (F): GGAGAAATGCGTGGATACCT
Axin2 (R): CTGCTTGGAGACAATGCGT
CD44 (F): CACCCCATCCCAGACGAAGA
CD44 (R): CCAGCCTGCTGAGATGGTATTTGA
c-myc (F): ACCACCAGCAGCGACTCTGA
c-myc (R): TCCAGCAGAAGGTGATCCAGACT
GAPDH (F): TCTCCTCTGACTTCAACAGC
GAPDH (R): GAAATGAGCTTGACAAAGTG

## Results

### β-catenin-dependent transcription is required for the induction of epithelial stem cell markers in CR HECs

β-catenin-dependent transcription maintains epithelial stem cell populations *in vivo* [8], and nuclear (active) β-catenin increases during conditional reprogramming of primary HECs to a stem cell-like state *in vitro* [3]. Therefore, we asked whether β-catenin-dependent transcription is required for the induction of epithelial stem cell markers in CR cells.

**Fig 1:**
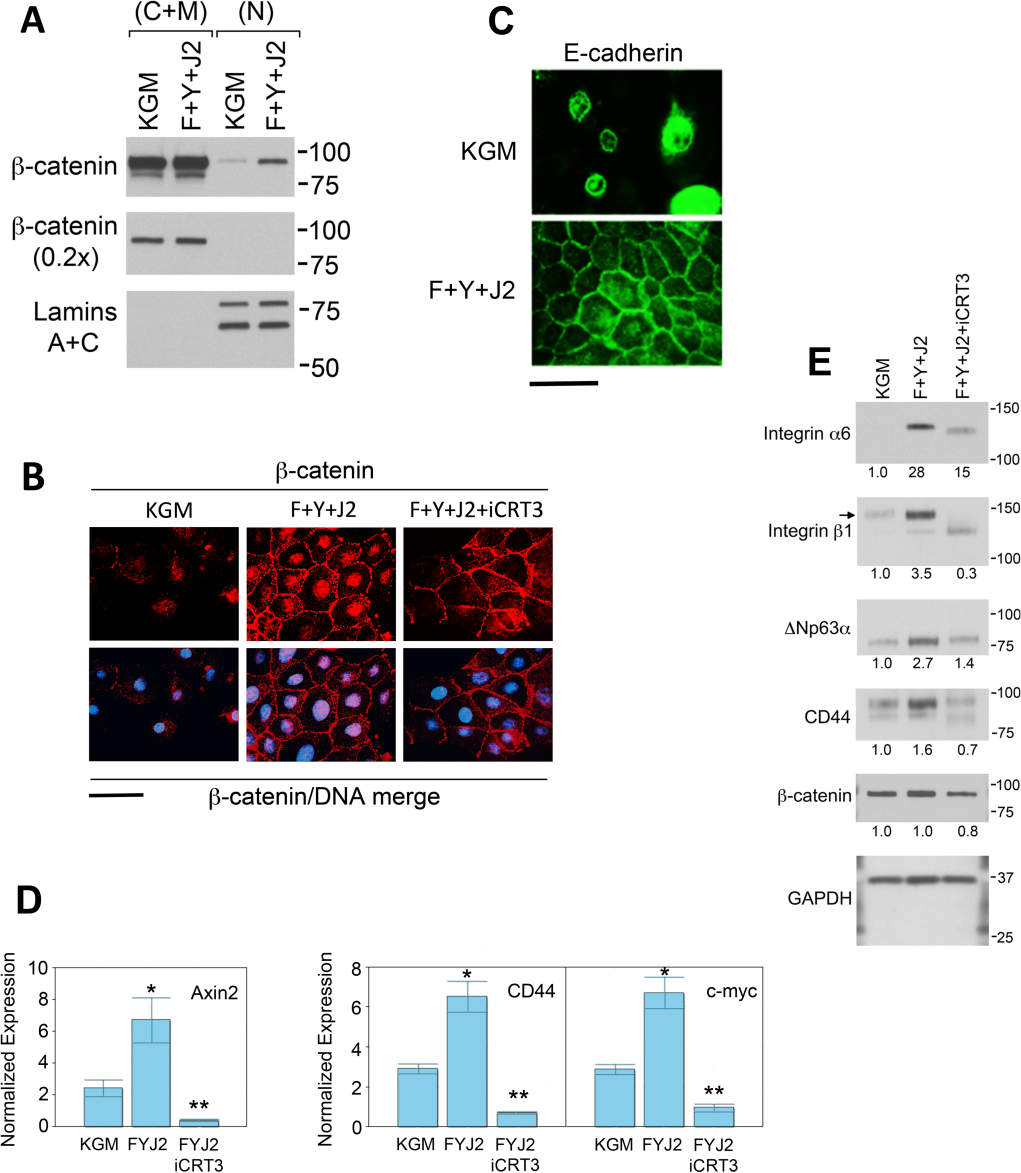
β-catenin-dependent transcription is required for increased expression of epithelial stem cell markers in CR HECs. (A) Western blots of nuclear (N) and non-nuclear (cytoplasm + membrane; C+M) fractions prepared from HECs maintained in KGM (KGM), or CR conditions (co-cultured with irradiated J2 fibroblasts in F-medium containing 10 μM Y-27632) for 3 d (F+Y+J2). All lanes were derived from equivalent numbers of cells. The lower (0.2x) β-catenin immunoblot was loaded with 1/5 as many cell equivalents as the upper. The efficiency of cell fractionation was verified by distribution of the nuclear scaffolding proteins, lamins A and C. (B) Primary HECs were cultured on coverslips for 3 d in KGM (KGM) or under CR conditions with (F+Y+J2+iCRT3) or without (F+Y+J2) 25 μM iCRT3. The HECs were extracted with TX-100, fixed and labeled with anti-β-catenin antibodies and Hoechst dye as described in Materials and Methods. Scale bar: 10 μm. (C) After 3 d in KGM (KGM) or CR culture (F+Y+J2), HECs on coverslips were fixed without prior detergent extraction and labeled with E-cadherin antibodies to visualize adherins junctions. Scale bar: 10 μm. (D) After 3 d in KGM (KGM), or CR cell culture with (FYJ2iCRT3) or without (FYJ2) 25 μM iCRT3, J2 cells were removed from 25-cm^2^ flasks by differential trypsin treatment and HECs processed for qRT-PCR analysis to determine levels of β-catenin-dependent mRNA transcripts. Error bars indicate standard deviation (S.D.) from the mean. (*) P-value < .00001 relative to KGM. (**) P-value < .00001 relative to FYJ2. (E) HECs were maintained for 3 d in KGM (KGM), or CR culture with (F+Y+J2+iCRT3) or without (F+Y+J2) 25 μM iCRT3 on 10-cm dishes. J2 cells were removed by EDTA treatment and whole-cell lysates of the HECs prepared for quantification of epithelial stem cell markers on Western blots. Lanes contain equal amounts of protein. Molecular mass markers (in kDa) are shown on the right. Arrow indicates Integrin β1 at 140 kD. The lower band at 120 kD appears to result from non-specific staining.

Consistent with our previous finding [3], cell fractionation demonstrated increased nuclear β-catenin in CR HECs (Fig 1A). However, at least 80% of total β-catenin was non-nuclear in both KGM and CR cultures (Fig 1A). Therefore, it was necessary to extract non-nuclear β-catenin using TX-100 prior to fixation to visualize nuclear β-catenin by immunofluorescence microscopy, which was increased in CR cells (Fig 1B). The localization of E-cadherin in CR cells also was indicative of adherins junctions, which were not present in KGM that essentially is a calcium-free medium (Fig 1C). Since β-catenin associates with E-cadherin in adherins junctions, peripheral β-catenin staining persisted in CR cells after detergent extraction (Fig 1B). This contrasted with KGM cultures, in which β-catenin was primarily soluble, and therefore susceptible to detergent extraction (Fig 1B).

In the nucleus, β-catenin associates with LEF/TCF family proteins to form a bipartite transcription factor [9]. The small molecule inhibitor, iCRT3, interferes with β-catenin binding to TCF and thereby inhibits β-catenin-dependent transcription without affecting its structural interactions with E-cadherin and α-catenin [17]. This specificity makes iCRT a much more suitable inhibitor than siRNA-or shRNA-mediated knockdown of β-catenin, which would interfere with all β-catenin functions and thereby potentially cloud interpretation of the results. Interestingly, because β-catenin was unable to bind LEF/TCF family proteins in the presence of 25 μM iCRT3 it was not retained in the nucleus in CR HECs (Fig 1B). The effectiveness of iCRT3 to inhibit transcription of three genes known to be regulated by β-catenin [18] is shown in Fig 1D. HECs conditionally reprogrammed for 3 d in co-culture with irradiated J2 fibroblasts and Y-27632 exhibited a 2-3-fold increase in Axin2, CD44 and c-myc mRNA relative to cells in KGM. However, inclusion of 25 μM iCRT3 during reprogramming reduced the abundance of these transcripts by approximately 85%, i.e. below their respective levels in KGM (Fig 1D). This concentration of iCRT3 is 3-fold lower than was used to inhibit β-catenin-dependent transcription in numerous normal and cancer cell lines [17].

As previously reported [3], conditional reprogramming increased expression of the epithelial stem cell markers integrin α6 (28-fold), integrin β1 (3.5-fold), ΔNp63α (2.7-fold) and CD44 (1.6-fold) in HECs (Fig 1E). However, 25 μM iCRT3 largely (integrin α6 and ΔNp63α) or completely (integrin β1 and CD44) prevented these increases with a minimal decrease in the overall level of β-catenin (Fig 1E). A glyceraldehyde 3-phosphate dehydrogenase (GAPDH) immunoblot demonstrated that all lysates contained equal amounts of protein (Fig 1E). Taken together, our results show that nuclear β-catenin increases in CR HECs and is necessary for up-regulated expression of epithelial stem cell markers.

### Wnt signaling does not activate β-catenin in CR HECs

**Fig 2:**
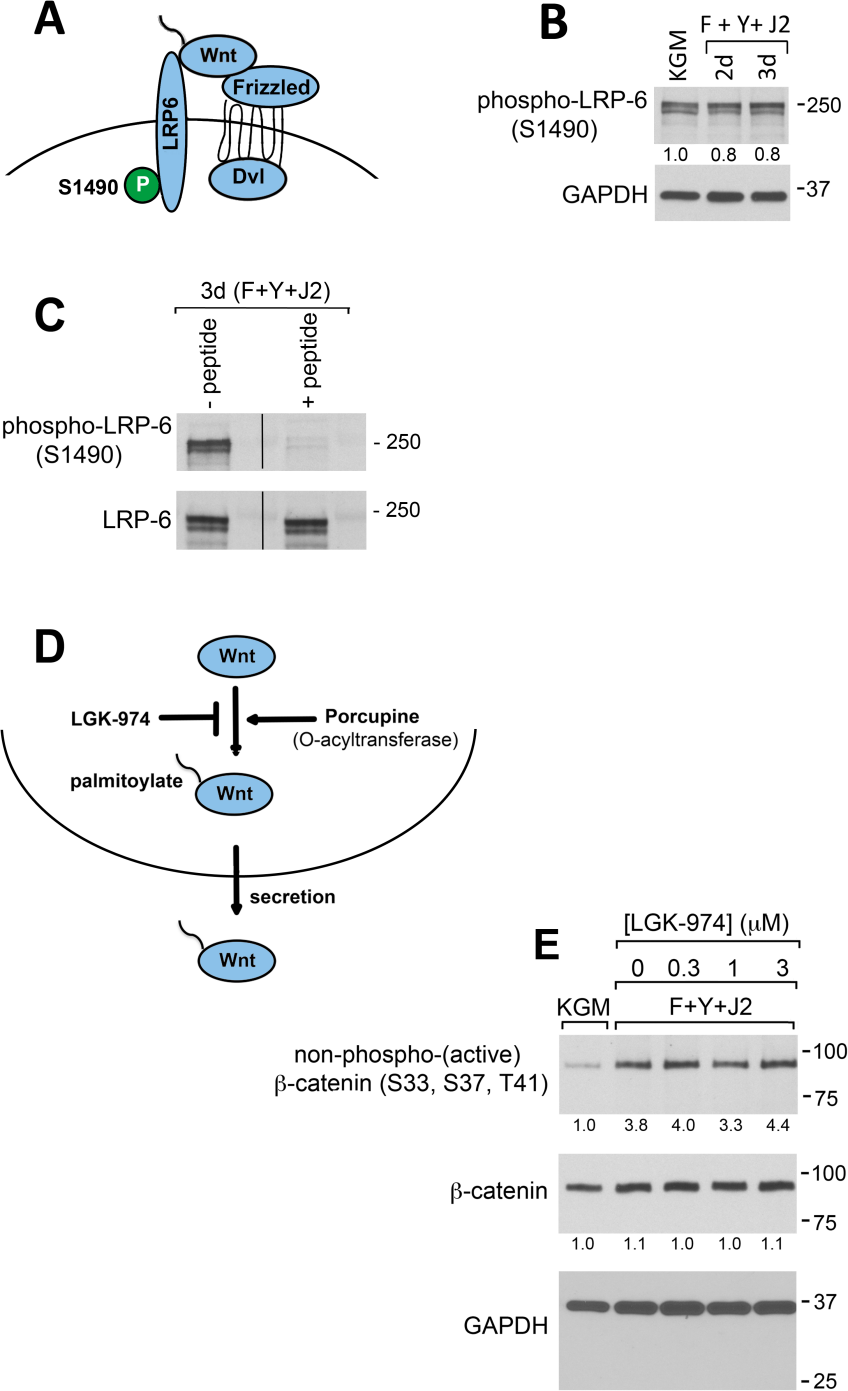
Wnt signaling does not activate β-catenin in CR HECs. (A) Diagram showing Wnt-dependent phosphorylation of LRP6 at S1490. (B) Western blot analysis of LRP6 phosphorylation at S1490 in whole-cell lysates prepared from HECs in KGM (KGM) after 3 d, or from 2–3 d CR cultures (F+Y+J2). All cultures included 0.15% DMSO. (C) Western blot of phospho-LRP6 (S1490) and total LRP6 from 3 d HEC CR cultures with and without pre-incubation of the primary antibody with a blocking peptide recognizing the S1490 phosphorylation site (see Materials and methods). (D) Schematic drawing showing LGK-974 inhibition of Wnt palmitoylation by Porcupine, which is obligatory for Wnt secretion. (E) Western blot analysis of β-catenin activation in whole-cell lysates prepared from HECs in 3 d KGM cultures (KGM), or from 3 d CR cultures in the presence of 0–3 μM LGK-974. All cultures included 0.15% DMSO. In (B), (C) and (E), lanes contain equal amounts of protein. Molecular mass markers (in kDa) are shown on the right.

Wnt ligands (Wnts) are a large family of secreted glycoproteins that control fundamental aspects of embryonic development and adult stem cell proliferation by regulating the stability of β-catenin [12]. Wnts bind to LRP5/6 and Frizzled co-receptors on the cell surface and recruit the axin complex, which includes GSK-3 and β-catenin. As a result, LRP6 is phosphorylated at multiple sites, including S1490 (Fig 2A), and β-catenin is activated [10]. As shown in Fig 2B, the level of LRP6 phosphorylated at S1490 did not change in HECs transitioned from KGM to CR co-culture, suggesting that β-catenin is not activated via Wnt signaling. The specificity of antibody recognition in this immunoblot was demonstrated by a strong reduction (80%) in labeling when the primary antibody was pre-incubated with a peptide corresponding to the LRP6 S1490 phospho-epitope (Fig 2C). In contrast, no reduction in labeling was observed when a primary antibody recognizing an LRP6 epitope distinct from the phosphorylation site was pre-incubated with the same peptide (Fig 2C).

Wnt secretion requires post-translational attachment of palmitoleic acid that is catalyzed by the membrane-bound O-acyltransferase, Porcupine [19,20]. LGK-974 is a potent and specific Porcupine inhibitor with an IC50 of 0.4 nM [21]. Therefore, addition of LGK-974 to CR cultures would be expected to inhibit Wnt secretion from both the irradiated J2 cells and HECs (Fig 2D). To confirm that Wnt signaling does not activate β-catenin in CR HECs, cultures were established in KGM, or under CR conditions in the presence of 0–3 μM LGK-974 and prepared for Western blot analysis 3 d later. The results (Fig 2E) showed that LGK-974 did not reduce levels of non-phospho (active) β-catenin and total β-catenin. Taken together with our analysis of LRP6 phosphorylation, these results show that Wnts do not activate β-catenin in CR HECs.

### GSK-3 is not inactivated as a consequence of conditional reprogramming in HECs

**Fig 3:**
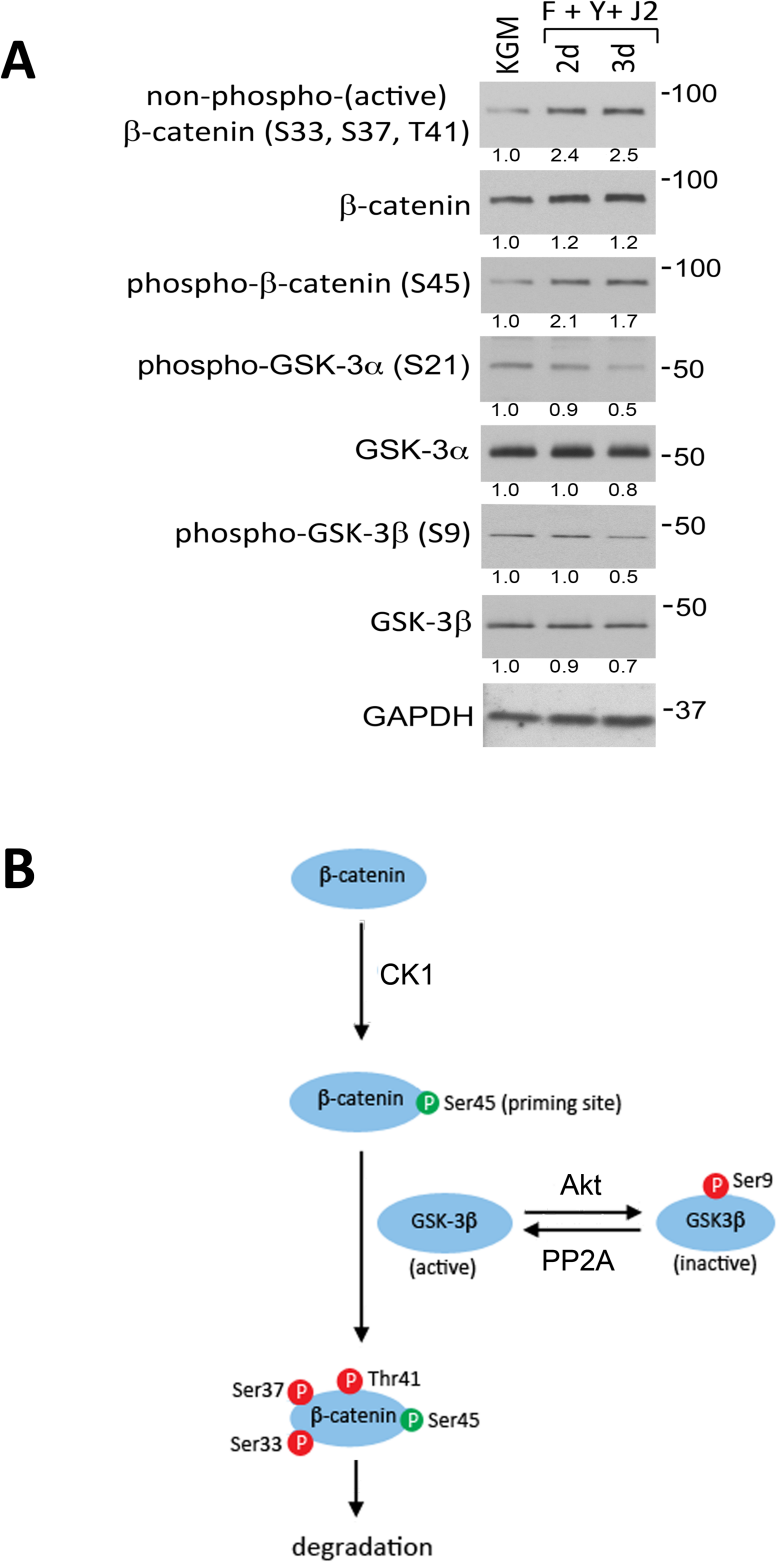
GSK-3 signaling during conditional reprogramming of HECs. (A) Western blot analysis of β-catenin and GSK-3 phosphorylation in whole-cell lysates prepared from HECs in KGM (KGM) after 3 d or after 2–3 d in CR culture (F+Y+J2). Phosphorylation sites are indicated in parenthesis. Lanes contain equal amounts of protein. Molecular mass markers (in kDa) are shown on the right. (B) Signaling pathway by which CK1, GSK-3, Akt and PP2A regulate β-catenin stability and activity. The β-catenin priming phosphorylation site at S45 is green; inhibitory and destabilizing phosphorylation sites are red.

The stabilization and nuclear accumulation of β-catenin is a direct consequence of dephosphorylation at 3 N-terminal sites (S33, S37 and T41), which prevents its ubiquitination and proteosomal degradation [10]. A monoclonal antibody that specifically recognizes β-catenin that is not phosphorylated at these sites (non-phospho β-catenin) showed that the level of non-phospho (active) β-catenin increased 2.5-fold during conditional reprogramming of HECs for 3 d (Fig 3A). In another experiment, the increase was as high as 3.8-fold (Fig 2E). The overall level of β-catenin did not increase significantly (Fig 3A), since only a small proportion underwent dephosphorylation and nuclear translocation (Fig 1A). The phosphorylation of β-catenin at S33, S37 and T41 is mediated by GSK-3 and requires prior “priming” phosphorylation of β-catenin at S45 by casein kinase 1 (CK1) (Fig 3B) [22]. S33/S37/T41 phosphorylation was not lower in CR HECs due to reduced priming, as phosphorylation at S45 actually increased 1.7-fold (Fig 3A).

Mammalian cells express two homologs of GSK-3, GSK-3α and GSK-3β, which are functionally redundant. Only cells in which three or four alleles are knocked out exhibit impaired β-catenin-dependent transcription [23]. GSK-3α and GSK-3β are inactivated by Akt-mediated phosphorylation at S21 and S9 respectively (Fig 3B) [24]. However, GSK-3 was not inhibited in CR HECs, since levels of both phospho-GSK-3α (S21) and phospho-GSK-3β (S9) actually decreased by 50% during reprogramming (Fig 3A). These data suggest that the activity of GSK-3 isoforms actually increases in CR HECs, perhaps as a result of AKT inhibition.

### Conditional reprogramming inhibits Akt signaling in HECs

**Fig 4:**
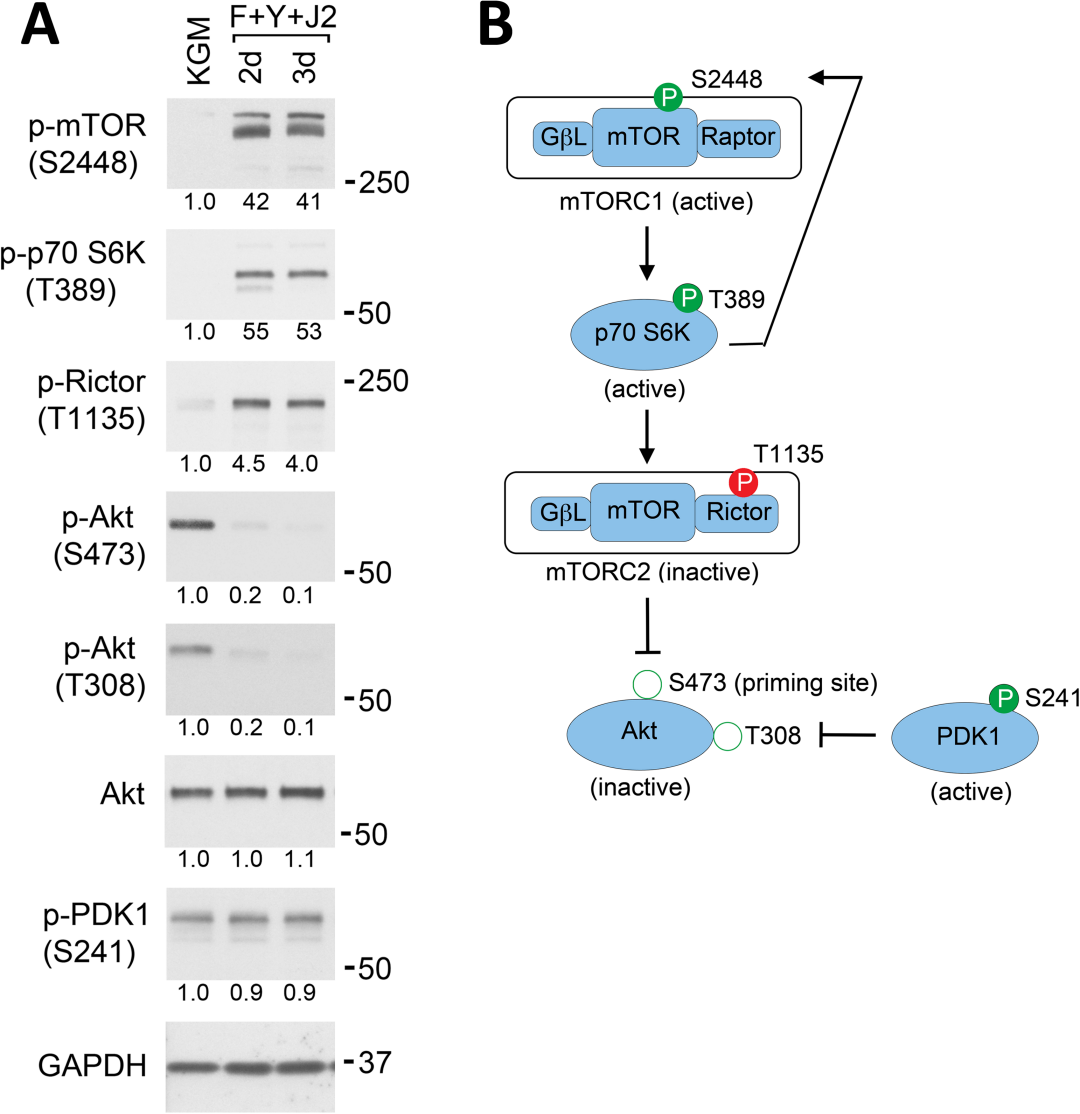
mTOR signaling inactivates Akt in CR HECs. (A) Western blot analysis of the mTOR signaling pathway in CR HECs. Whole-cell lysates were prepared from 3 d cultures of HECs in KGM (KGM), or from 2–3 d CR cultures (F+Y+J2). Phosphorylation sites are indicated in parenthesis. Lanes contain equal amounts of protein. Molecular mass markers (in kDa) are shown on the right. (B) Diagram of mTORC1/Akt signaling in CR cells. Activating phosphorylation sites are shown as filled green circles and inhibitory phosphorylation sites as filled red circles. Phosphorylation of the mTORC1 complex at S2448 by p70 S6K is indicative of active mTORC1. Hollow green circles show activating sites on Akt that exhibit dramatically reduced phosphorylation in CR HECs.

The Akt protein kinase promotes cell proliferation and survival by phosphorylating a myriad of downstream targets, including GSK-3α and GSK-3β [25]. Since CR cells proliferate rapidly and can be propagated indefinitely, it was noteworthy that the phosphorylation of Akt sites in GSK-3α and GSK-3β (S21 and S9 respectively) decreased by 50% in CR HECs (Fig 3A). The activation of Akt requires phosphorylation at two sites: a “priming” phosphorylation at S473, catalyzed by the mammalian target of rapamycin complex 2 (mTORC2), followed by phosphoinositide-dependent kinase 1 (PDK1)-mediated phosphorylation at T308 [26]. A Western blot of HECs transitioned from KGM to CR conditions for 2–3 d showed that PDK1 kinase activity did not change, as evidenced by a constant level of autophosphorylation at S241 (Fig 4A) [27]. However, Akt phosphorylation decreased by 90% at T308 and S473 (Fig 4A), indicating reduced Akt kinase activity. This result was explained by a 4-fold increase in Rictor phosphorylation at T1135 (Fig 4A). Rictor is a component of the mTORC2 complex that phosphorylates the Akt priming site, and Rictor phosphorylation at T1135 is known to inhibit mTORC2 kinase activity *in vivo* [28]. Rictor T1135 in turn is phosphorylated by the p70 S6 kinase (p70 S6K) [25], which is activated by phosphorylation at T389 by the mammalian target of rapamycin complex 1 (mTORC1) [29,30]. As shown in Fig 4A, p70 S6K phosphorylation at T389 increased 53-fold in conditionally reprogrammed HECs, indicating that the activity of both p70 S6K and mTORC1 were strongly up regulated. Phosphorylation of the mTOR component of mTORC1 at S2448 also is mediated by p70 S6K [27], and increased 41-fold (Fig 4A). Therefore, mTOR signaling is activated in CR HECs, which dramatically reduces Akt activity (Fig 4B).

### A major role for PP2A in the conditional reprogramming of HECs

**Fig 5:**
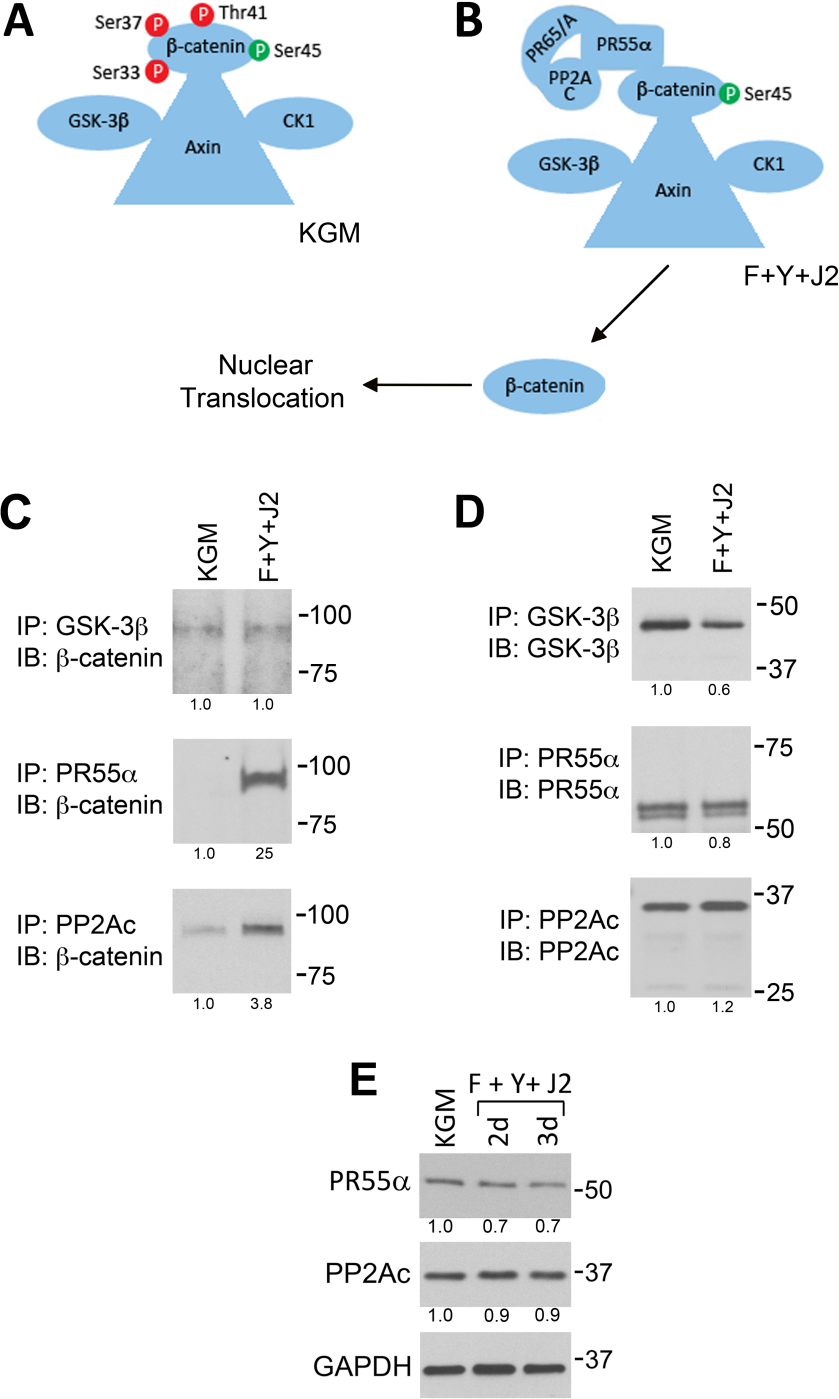
Increased association of PP2A regulatory and catalytic subunits with β-catenin in CR HECs. (A) Graphic illustration of the β-catenin complex in KGM. (B) Illustration of the β-catenin complex in CR cells (F+Y+J2). In (A) and (B), the β-catenin priming phosphorylation site at S45 is green, while destabilizing phosphorylation sites are red. (C) GSK-3β, PR55α and PP2Ac were immunoprecipitated (from equal amounts of protein) from HECs in KGM cultures (KGM), or from CR cultures (F+Y+J2) after 3 d. The immunoprecipitates (IP) were analyzed on immunoblots (IB) labeled for β-catenin to measure association between the proteins. (D) Control experiments (IP and IB the same protein) verified that equivalent amounts of each protein were immunoprecipitated in (C). (E) Total levels of PR55α and PP2Ac in 3 d KGM cultures (KGM) and in HECs conditionally reprogrammed for 2–3 d (F+Y+J2). Lanes contain equal amounts of protein. In (C), (D) and (E) molecular mass markers (in kDa) are shown on the right.

Outside of the nucleus, β-catenin is either bound to E-cadherin at the plasma membrane or to the scaffolding protein axin in a complex that includes CK1 and GSK-3 (Fig 5A) [31]. GSK-3 phosphorylates β-catenin at sites that promote proteosomal degradation (S33, S37 and T41) [10]. PP2A is a serine/threonine protein phosphatase that dephosphorylates β-catenin at S33, S37 and T41 [10] and is a heterotrimer comprised of structural (A), regulatory (B, B’, B” or B”‘) and catalytic (C) subunits. The regulatory subunit is responsible for targeting PP2A to specific substrates and is by far the most diverse [32]. The α isoform of the 55 kDa B-type regulatory subunit, PR55α, binds directly to β-catenin and targets it for dephosphorylation by the PP2A catalytic subunit (PP2Ac) (Fig 5B) [11].

Since canonical Wnt and Akt signaling pathways did not explain the activation of β-catenin in CR HECs, we used immunoprecipitation and Western blotting to quantify direct and indirect associations between GSK-3, PP2A and β-catenin in HECs cultured in KGM, or after 3 d of conditional reprogramming. While the amount of β-catenin that co-precipitated with GSK-3β was identical in both culture conditions, 25-fold more β-catenin co-precipitated with PR55α in CR cells (Fig 5C). In addition, 3.8-fold more β-catenin co-precipitated with PP2Ac (Fig 5C). Control immunoblots (Fig 5D) showed that similar amounts of GSK-3β, PR55α and PP2Ac were immunoprecipitated in both culture conditions (GSK-3β and PR55α were reduced by 20% and PP2Ac was increased by 20% relative to KGM). Increased PP2A binding to β-catenin was observed despite a 30% reduction in total PR55α and 10% reduction in total PP2Ac (Fig 5E). Therefore, β-catenin activation could not be attributed to increased expression of PP2A subunits. These results demonstrate increased association between PP2A and β-catenin in CR HECs and suggest a PP2A-dependent mechanism by which β-catenin is activated.

### Okadaic acid inhibits β-catenin activation and the induction of epithelial stem cell markers

**Fig 6:**
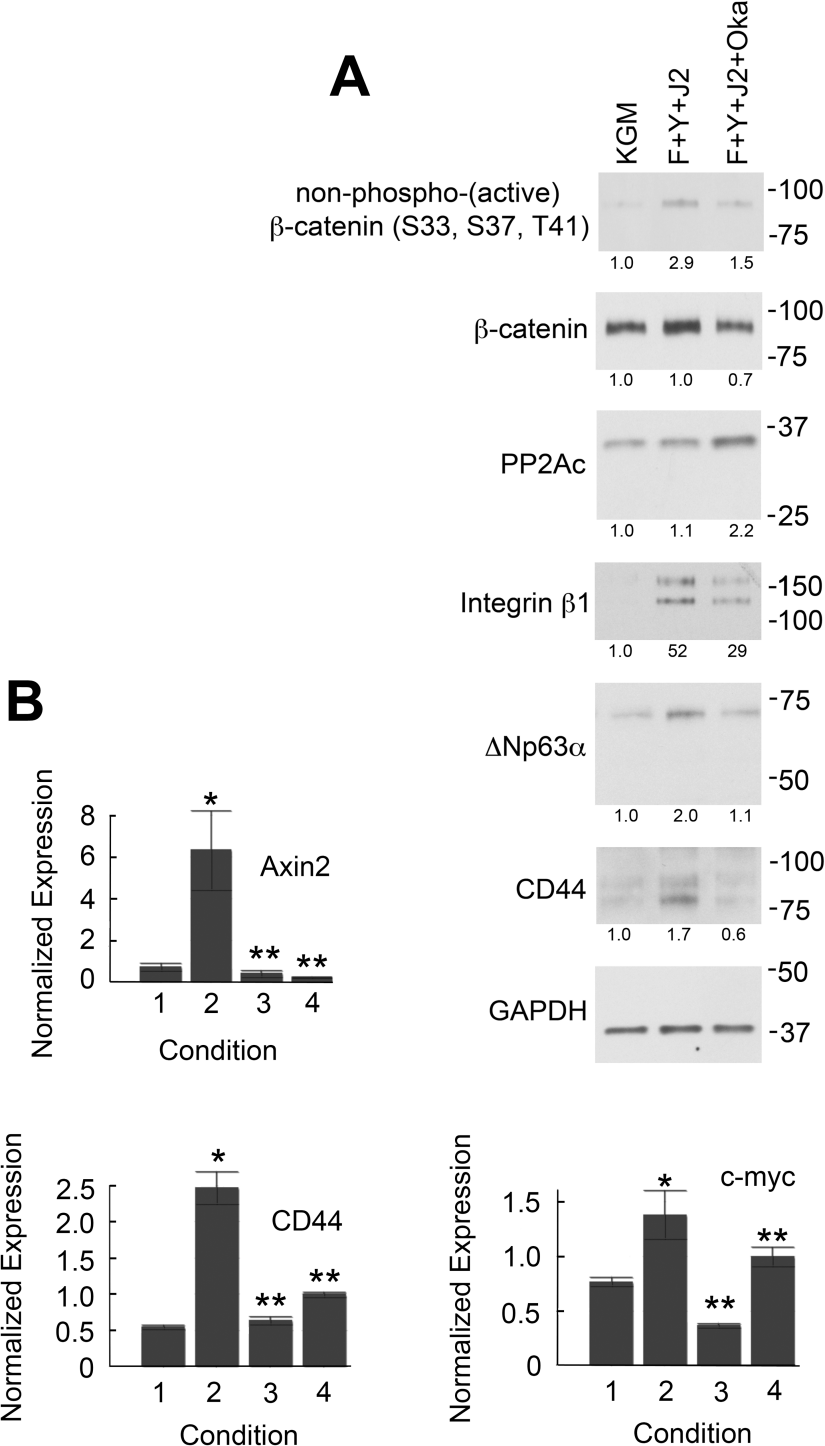
OA inhibits both β-catenin activation and the induction of epithelial stem cell markers. (A) HECs were cultured for 3 d in KGM (KGM), or in CR conditions (F+Y+J2). In (F+Y+J2+Oka), 10 nM OA was added during the final 24 h of conditional reprogramming. Whole-cell lysates were then analyzed by Western blotting to determine levels of active β-catenin, total β-catenin and epithelial stem cell markers. Molecular mass markers (in kDa) are shown on the right. Lanes contain equal amounts of protein. (B) OA treatment inhibits β-catenin-dependent transcription. After 3 d in KGM (condition1), or in CR cell culture without OA (condition 2) J2 cells were removed from 60-mm dishes by differential trypsin treatment and HECs processed for qRT-PCR analysis to determine levels of β-catenin-dependent mRNA transcripts. HECs were similarly processed in conditions 3 and 4, except that the CR cultures were treated with 10 nM OA for the final 6 h (condition 3) or 24 h (condition 4) before lysis. Error bars indicate standard deviation (S.D.) from the mean. (*) P-value < .00001 relative to condition 1 (KGM). (**) P-value < .00001 relative to condition 2 (CR culture without OA).

Okadaic acid (OA) is a potent and selective inhibitor of protein phosphatase 1 (PP1) and PP2A *in vitro* and *in vivo* [33]. To support the hypothesis that increased association with PP2A activates β-catenin in CR cells, HECs were conditionally reprogrammed for 3 d with or without addition of 10 nM OA for the final 24 h. This concentration of OA completely inhibits PP2A-mediated dephosphorylation of Erk 1 and Erk 2 in primary human keratinocytes [34]. In contrast, > 1 μM OA is required to inhibit PP1 *in vivo* [34]. As shown (Fig 6A), 3 d of conditional reprogramming increased levels of non-phospho (active) β-catenin 2.9-fold relative to KGM without increasing total β-catenin. However, OA treatment reduced the level of non-phospho β-catenin by 50% (Fig 6A). To demonstrate that OA inhibited β-catenin-dependent transcription, qRT-PCR was used to measure levels of Axin2, CD44 and c-myc mRNAs in CR HECs with and without OA treatment. As previously shown (Fig 1D), levels of mRNA encoding these proteins increased 2-6-fold when HECs were transferred from KGM to CR conditions for 3 d (Fig 6B, condition 2 vs. 1). However, treatment with 10 nM OA for 6 h prior to lysis of the CR cells reduced transcript levels by 74–93% (Fig 6B, condition 3), while treatment for 24 h reduced levels by 28–96% (Fig 6B, condition 4). The observation that treatment with OA for 6 h was more effective at repressing β-catenin-dependent transcription than treatment for 24 h suggests that CR cells somehow compensate for the loss of PP2A activity over time. Therefore, the ability of OA to acutely inhibit PP2A makes it superior to siRNA- or shRNA-mediated knockdown of PP2A, which would require at least 48 h.

Since β-catenin-dependent transcription was required to up-regulate expression of epithelial stem cell markers in CR HECs (Fig 1E), OA inhibition of β-catenin activation might also be expected to inhibit the induction of stem cell markers. Relative to HECs cultured in KGM, integrin β1 expression increased 52-fold after 3 d in CR culture but only increased 29-fold when 10 nM OA was present during the final 24 h of reprogramming (Fig 6A). (6 h OA treatment was considered to be insufficient for protein expression analysis given the 23 h half-life of integrin β1 [35]). ΔNp63α expression increased 2.0-fold without OA, but only 1.1-fold when it was present. Similarly, CD44 expression increased 1.7-fold in the absence of OA, compared to 0.8-fold when it was present (Fig 6A). Therefore, OA treatment decreased levels of both non-phospho (active) β-catenin and epithelial stem cell markers to the same extent (50%). These results provide evidence that PP2A-mediated activation of β-catenin is important for the conditional reprogramming of primary HECs to an adult stem cell-like state.

### β-catenin activation and Akt inactivation in CR prostate and breast cells

**Fig 7:**
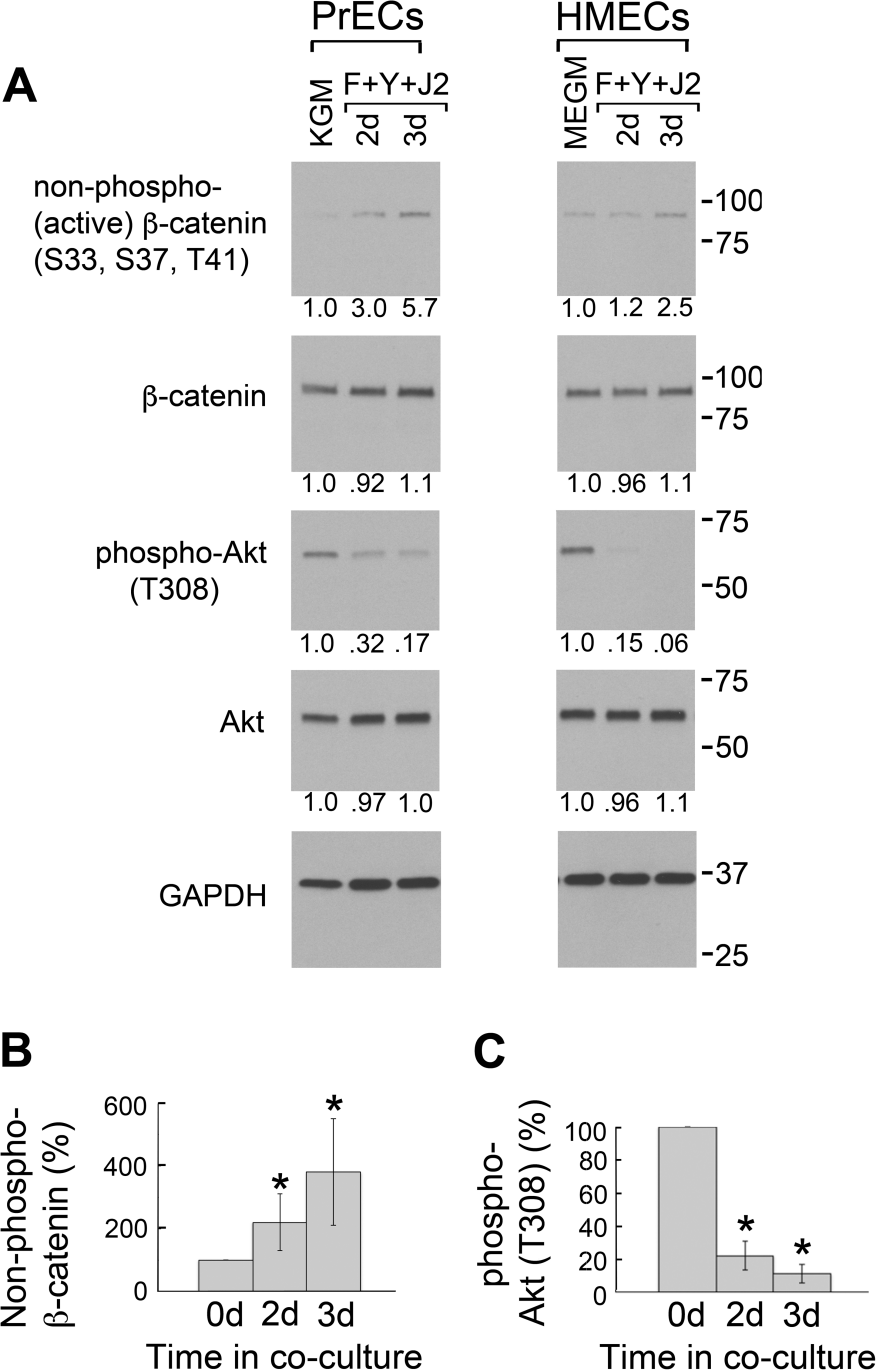
β-catenin activation and Akt inactivation in CR prostate and breast cells. (A) Levels of active (non-phospho S33, S37, T41) β-catenin and active (phospho-T308) Akt were quantified in PrECs and HMECs using Western blots of whole-cell lysates prepared from cells in synthetic media for 3 d (KGM for PrECs and MEGM for HMECs), or in co-culture with irradiated J2 fibroblasts and Y-27632 for 2–3 d (F+Y+J2). Lanes contain equal amounts of protein. Molecular mass markers (in kDa) are shown on the right. (B) Mean levels of active (non-phospho S33, S37, T41) β-catenin in HECs, PrECs and HMECs. Error bars indicate standard deviation (S.D.) from the mean. (*) P-value < .00001 relative to 0 d. (C) Mean levels of active (phospho-T308) Akt in HECs, PrECs and HMECs. Error bars indicate standard deviation (S.D.) from the mean. (*) P-value < .00001 relative to 0 d.

Very little is known about the biology of CR cells, however our previous [3] and present studies show that conditional reprogramming activates β-catenin and inactivates Akt in HECs. To determine if these characteristics extend to other cell types, whole-cell lysates were prepared from PrECs and HMECs in synthetic media (KGM for PrECs and MEGM for HMECs) and after 2–3 d of conditional reprogramming. As shown (Fig 7A), non-phospho (active) β-catenin increased 5.7-fold in CR PrECs and 2.5-fold in CR HMECs. In contrast, levels of total β-catenin did not change (1.1-fold increase in both cell types). Active Akt is characterized by phosphorylation at T308 [26]. While the total level of Akt did not change in CR PrECs and CR HMECs, levels of phospho-Akt (T308) decreased by 83% in CR PrECs and 94% in CR HMECs (Fig 7A). Taken together with our findings in CR HECs, these results suggest that that β-catenin activation and Akt inactivation may generally be attributes of the CR phenotype (Fig 7B and 7C).

### β-catenin phosphorylation at S675 may contribute to increased association with PP2A in CR cells

**Fig 8:**
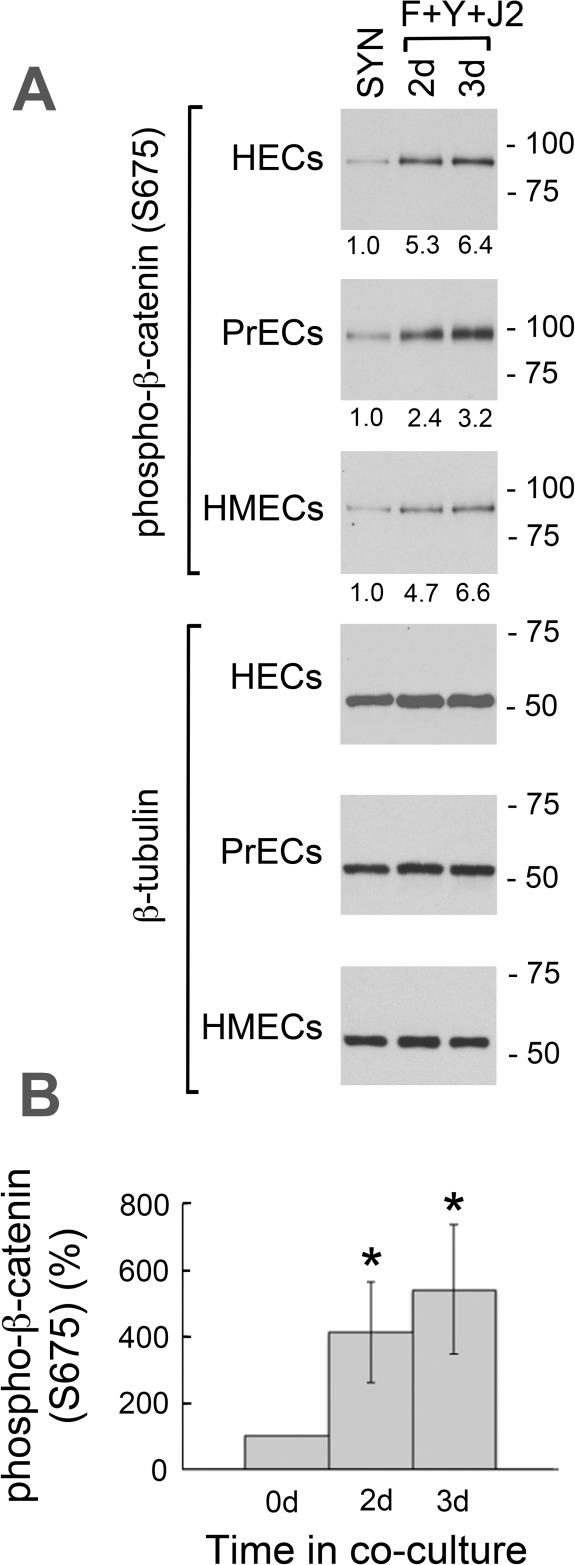
β-catenin phosphorylation at S675 in synthetic media and CR cultures. (A) Whole-cell lysates were prepared from 3 d cultures of HECs, PrECs and HMECs in synthetic media (SYN: KGM for HECs and PrECs; MEGM for HMECs), or from 2–3 d CR cultures (F+Y+J2). Western blots were labeled for phospho-β-catenin (S675) and normalized relative to identical immunoblots labeled for β-tubulin. Lanes contain equal amounts of protein. Molecular mass markers (in kDa) are shown on the right. (B) Mean phospho-β-catenin (S675) levels in the 3 cell lines. Error bars indicate standard deviation (S.D.) from the mean. (*) P-value < .00001 relative to 0 d.

β-catenin is stabilized as a result of reduced ubiquitination when phosphorylated at S675 by cAMP-dependent protein kinase (PKA) [36]. Since F-medium contains cholera toxin [3], which is known to activate PKA *in vivo* [37], we examined β-catenin (S675) phosphorylation on Western blots of HEC, PrEC and HMEC whole-cell lysates prepared from cultures in synthetic media, or from cultures transitioned to CR conditions for 2–3 d. As shown (Fig 8A), S675 phosphorylation increased 6.4-fold in HECs, 3.2-fold in PrECs and 6.6-fold in HMECs after 3 d in CR culture. The mean increase for all cell lines transitioned to CR conditions was 4.1-fold after 2 d and 5.4-fold after 3 d (Fig 8B). An increase in the stable pool of β-catenin would enable increased association with PP2A, followed by increased β-catenin activation as a result of PP2A-mediated dephosphorylation of β-catenin at S33, S37 and T41.

## Discussion

We previously showed that β-catenin is activated during the conditional reprogramming of primary HECs to an adult stem cell-like state, which is characterized by increased expression of epithelial stem cell markers [3] and indefinite proliferation *in vitro* [1]. In the present study we show that β-catenin also is activated in CR prostate and breast cells, and that β-catenin-dependent transcription is necessary for the increased expression of stem cell markers in CR HECs. iCRT3, a small molecule inhibitor that interferes with β-catenin binding to TCF to form a functional bipartite transcription factor, significantly reduces the level of β-catenin-dependent transcripts and the expression of stem cell markers. Moreover, we show that canonical Wnt and Akt/GSK-3 signaling pathways do not activate β-catenin in CR cells: LGK-974, a potent inhibitor of Wnt secretion, does not inhibit β-catenin activation, and Akt signaling (which inactivates GSK-3) is dramatically reduced in CR HECs, PrECs and HMECs. Rather, we demonstrate increased association of β-catenin with PP2A, and show that PP2A catalytic activity is important for β-catenin activation, β-catenin-dependent transcription and the increased expression of epithelial stem cell markers.

The ubiquitination and subsequent proteolysis of β-catenin is inhibited by PKA-mediated phosphorylation at S675 [36]. We show that the level of β-catenin phosphorylated at S675 increases 3.2–6.6-fold in CR HECs, PrECs and HMECs relative to cultures in synthetic media. This increase probably is due to the presence of cholera toxin in CR culture medium [3], which is known to activate PKA [37]. PKA-mediated stabilization of β-catenin in CR cultures would likely make more β-catenin available to bind PP2A, and therefore contribute to our observed increase in β-catenin-PP2A association. Nevertheless, it is noteworthy that β-catenin activation would still require PP2A-mediated dephosphorylation at S33, S37 and T41.

Since the Akt protein kinase generally promotes cell proliferation and survival, it was initially somewhat surprising that Akt signaling was reduced in CR cells, which proliferate rapidly and can be propagated indefinitely. However, Akt activity increases during keratinocyte differentiation [38,39] and transduction of mouse primary keratinocytes with a constitutively active Akt mutant induces growth arrest and differentiation [38]. Similarly, RNAi-mediated knockdown of Akt in primary human keratinocytes disrupts differentiation in organotypic raft cultures [39]. Therefore, low Akt activity seems to be important for maintaining epithelial stem cells in an undifferentiated, proliferative state.

Mechanistically, we determined that the phosphorylation of S473, a critical priming site on Akt, is greatly reduced in CR HECs due to inactivation of the corresponding kinase, mTORC2, by mTORC1 signaling. The reason for increased mTORC1 signaling is unknown, but several studies provide clues. mTORC1 is activated by direct association with the GTP-bound form of the small GTPase, Rheb [40]. The ratio of GTP-Rheb to GDP-Rheb is controlled by the Rheb GTPase activating protein (Rheb GAP), which is a heterodimer of hamartin (TSC1) and tuberin (TSC2). An active TSC1/TSC2 complex shifts this ratio in favor of GDP-Rheb, which cannot activate mTORC1 [41]. Assembly of the TSC1/TSC2 complex is promoted by phosphorylation of TSC2 [42]. Since the ROCK inhibitor, Y-27632, is essential for conditional reprogramming, it is significant that both rat [40] and human [41] TSC2 contain a consensus ROCK1 phosphorylation site (R/KXXS/T) at T1203. Rat TSC2 is phosphorylated at T1203 by recombinant ROCK1 *in vitro*, and substitution of alanine for T1203 severely impairs the ability of ROCK to inactivate mTORC1 *in vivo* [43]. When P1202 is mutated to histidine in human TSC2, Rheb GAP activity is lost and mTORC1 signaling is activated [44]. Even though the P1202H mutation maintains a R/KXXS/T motif, proline locally restricts protein flexibility more than histidine and therefore may influence the phosphorylation of neighboring serine and threonine residues [45].

Our finding that increased β-catenin-dependent transcription is important for the conditional reprogramming of primary human epithelial cells to an adult stem-like state is consistent with the role β-catenin plays in maintaining self-renewal in isolated adult stem cells. The expansion of mammary [46] and neural [47] stem cells is enhanced by the addition of Wnt (a β-catenin activator) to the culture medium, and overexpression of active β-catenin stimulates the proliferation of hematopoietic stem cells *in vitro* [48]. Modest or short-term activation of β-catenin by the addition of Wnt3a [49] or GSK-3β inhibitors [50] maintains self-renewal in mouse and human ESCs. However, high levels of transcriptionally active β-catenin [51], as occur in GSK-3β double-knockout cells [52], induce differentiation. Transient expression of Wnt2 also increases the efficiency of reprogramming mouse embryonic fibroblasts to iPS cells [53].

It has been reported that the inhibition of PP2A by OA promotes self-renewal in human ESCs [54], in contrast to its inhibitory effect on the induction of adult stem cell markers in CR cells. We have shown that conditional reprogramming involves increased β-catenin-dependent transcription, which coincides with increased PP2A binding to β-catenin. The observation that OA inhibits both β-catenin-dependent transcription and the expression of adult stem cell markers in CR cells emphasizes the importance of PP2A catalytic activity for conditional reprogramming. OA may affect ES and CR cells differently, since CR cells do not express the ESC markers Sox2, Oct4, and Nanog [3], and therefore represent a fundamentally different phenotype.

## Supporting information

S1 Fig Uncropped blots for Fig 1. (TIF)

S2 Fig Uncropped blots for Fig 2. (TIF)

S3 Fig Uncropped blots for Fig 3. (TIF)

S4 Fig Uncropped blots for Fig 4. (TIF)

S5 Fig Uncropped blots for Fig 5. (TIF)

S6 Fig Uncropped blots for Fig 6. (TIF)

S7 Fig Uncropped blots for Fig 7. (TIF)

S8 Fig Uncropped blots for Fig 8. (TIF)
