# Supplementary figures and images for: Conditional cell reprogramming involves non-canonical β-catenin activation and mTOR-mediated inactivation of Akt
Source: PLoS One. 2017 Jul 10;12(7):e0180897. doi: 10.1371/journal.pone.0180897 (PMC5507294; doi:10.1371/journal.pone.0180897)

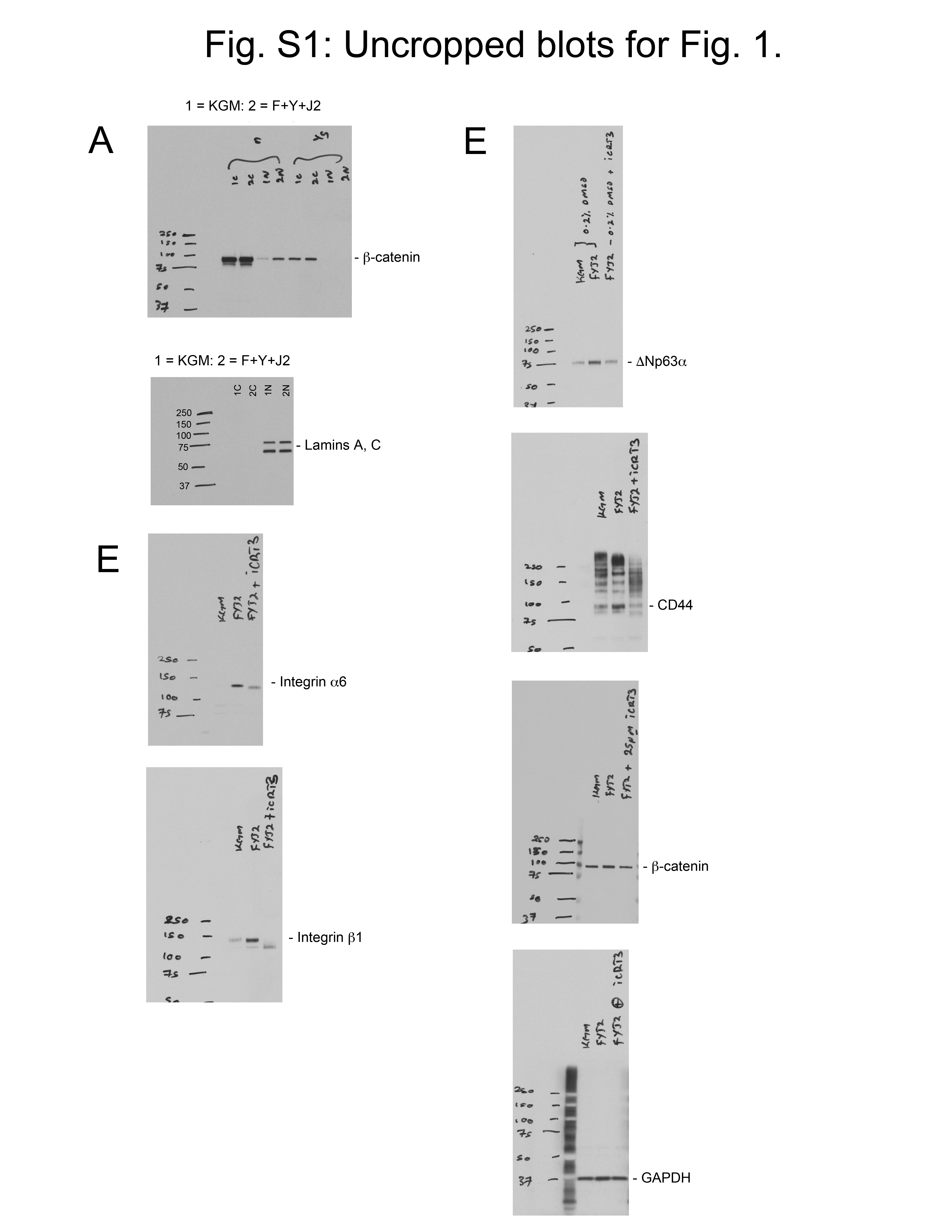

Supplement: S1 Fig — (TIF) [file pone.0180897.s001.tif]

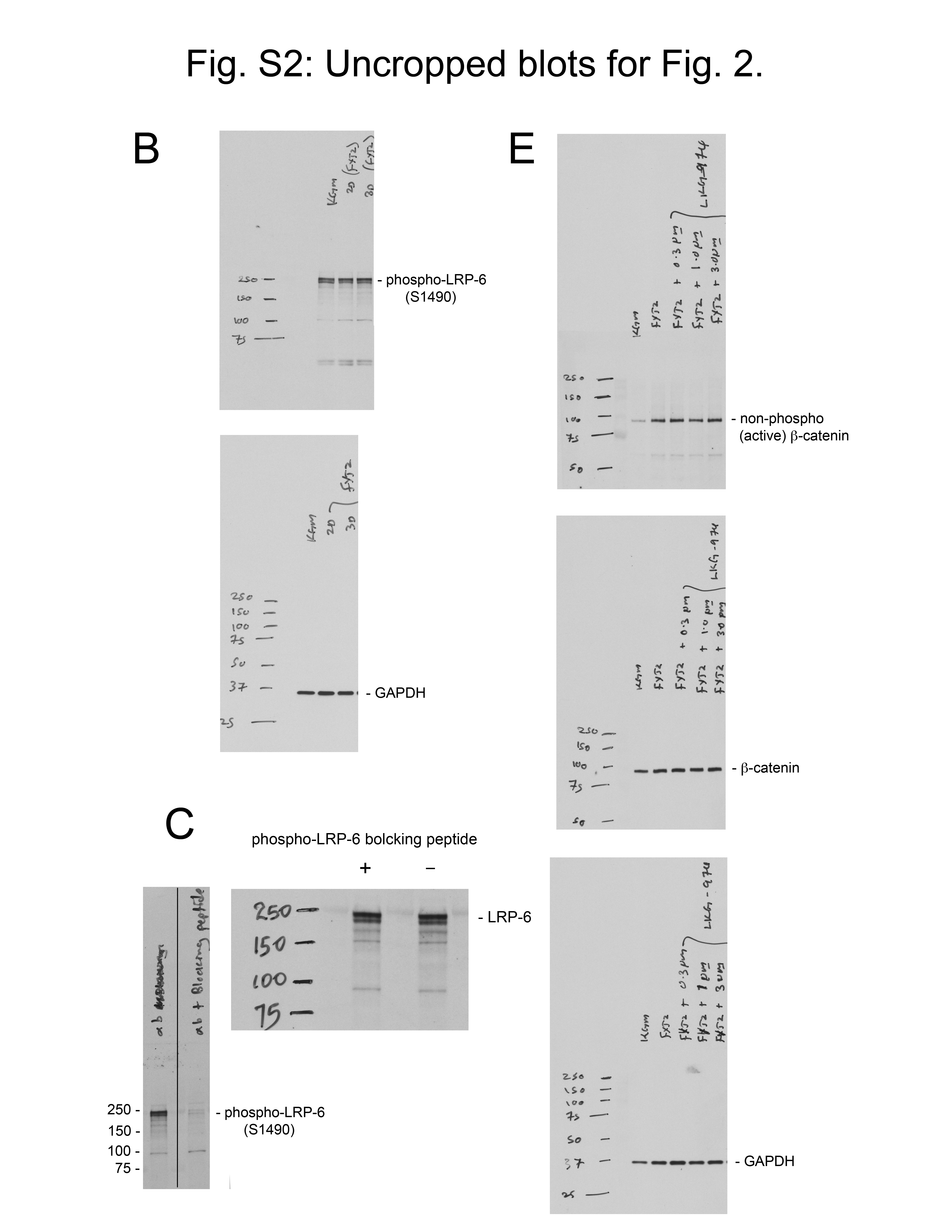

Supplement: S2 Fig — (TIF) [file pone.0180897.s002.tif]

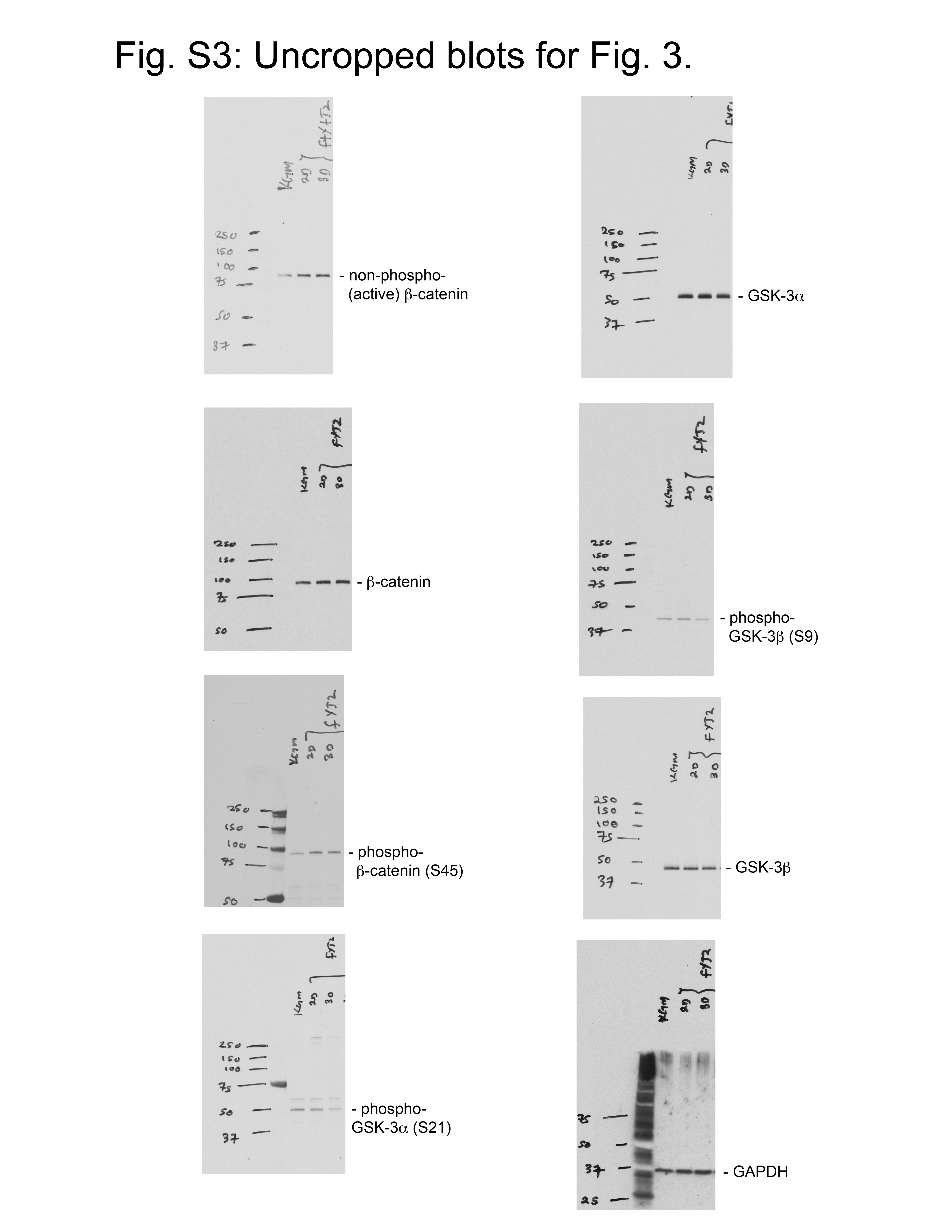

Supplement: S3 Fig — (TIF) [file pone.0180897.s003.tif]

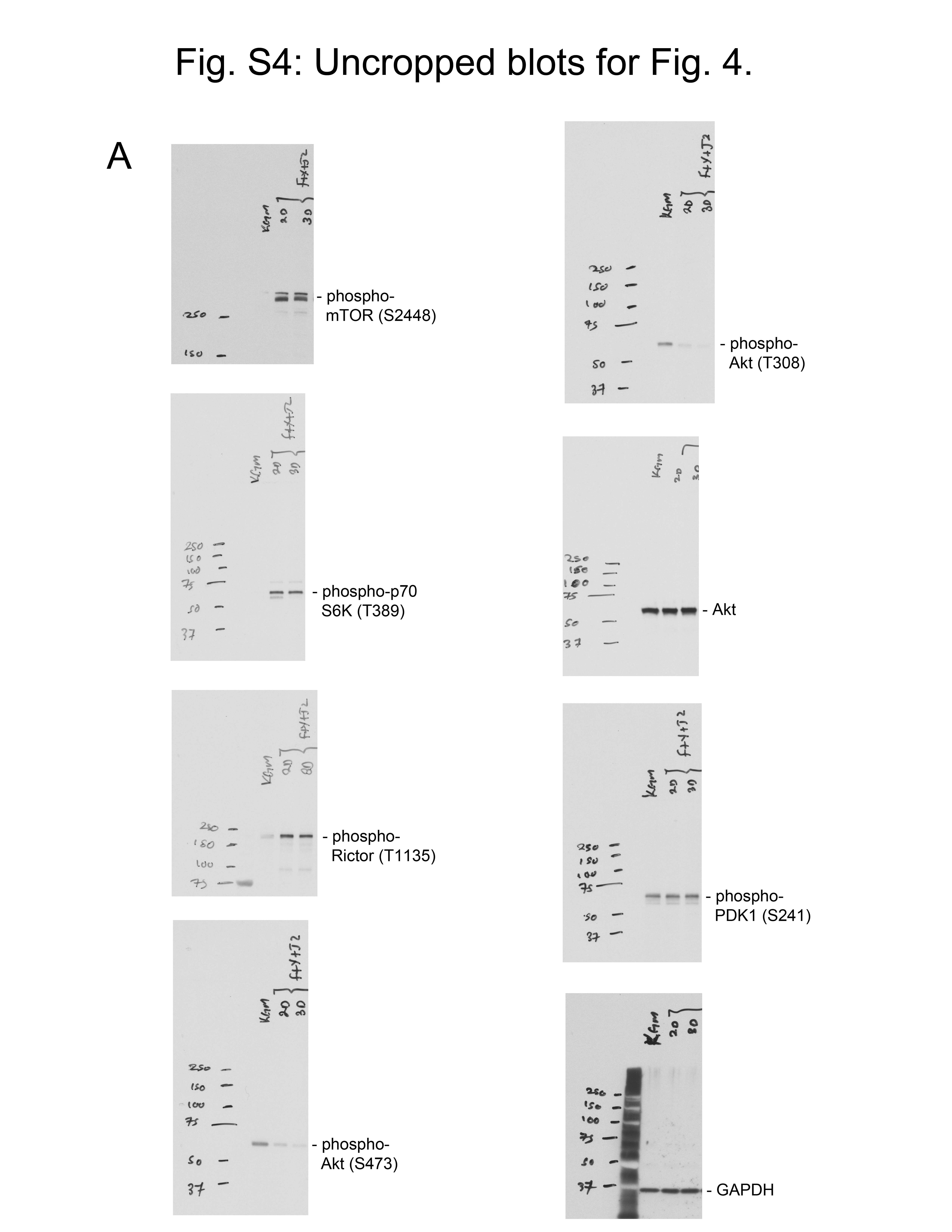

Supplement: S4 Fig — (TIF) [file pone.0180897.s004.tif]

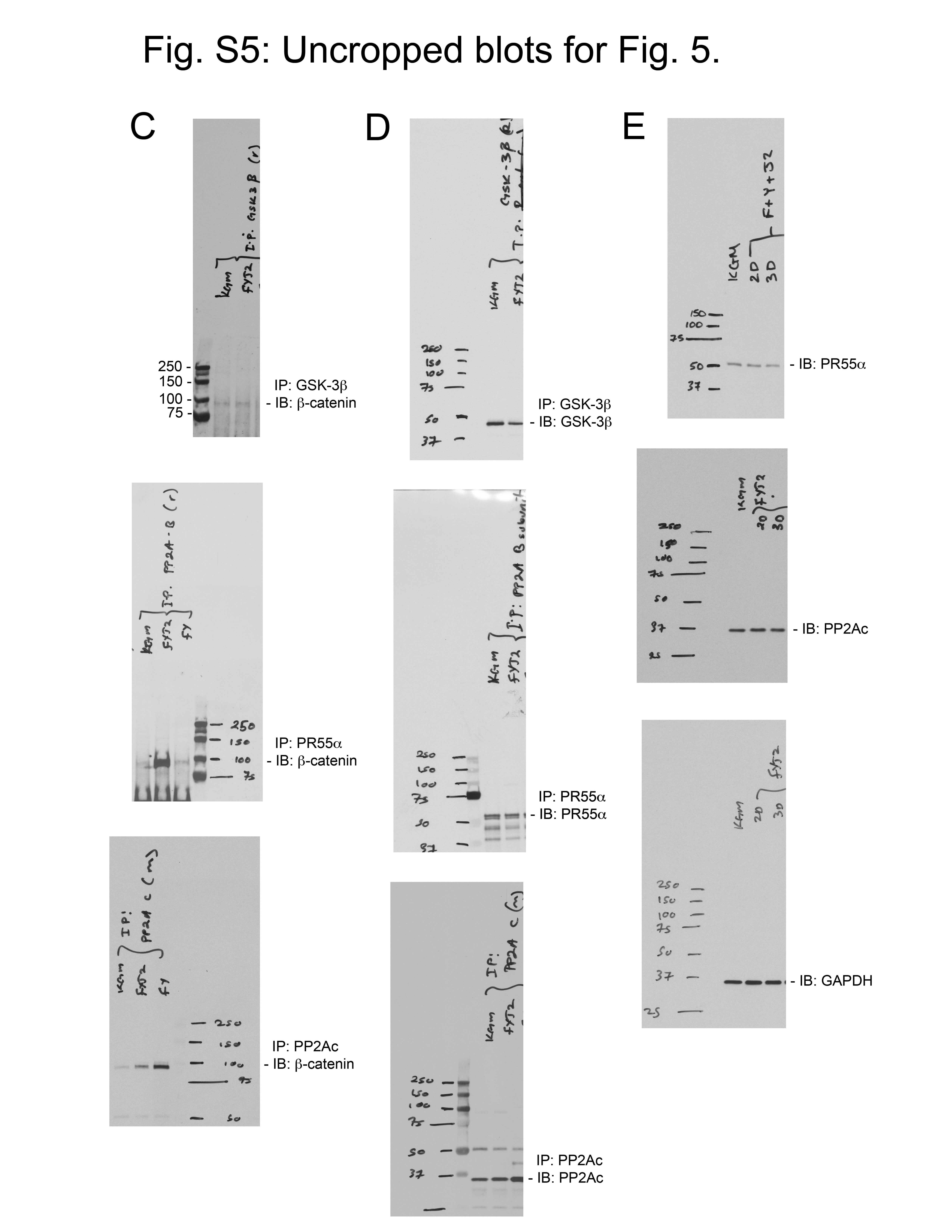

Supplement: S5 Fig — (TIF) [file pone.0180897.s005.tif]

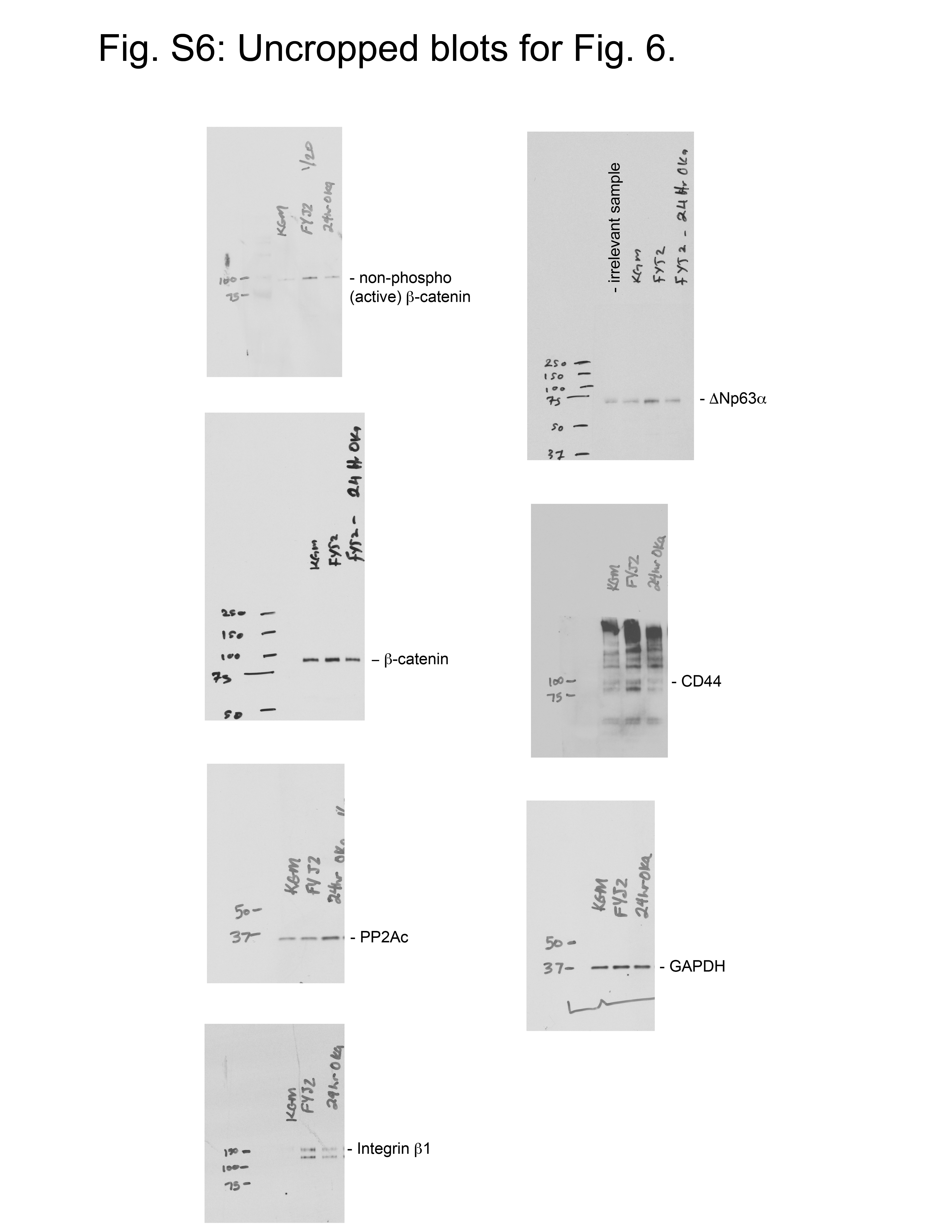

Supplement: S6 Fig — (TIF) [file pone.0180897.s006.tif]

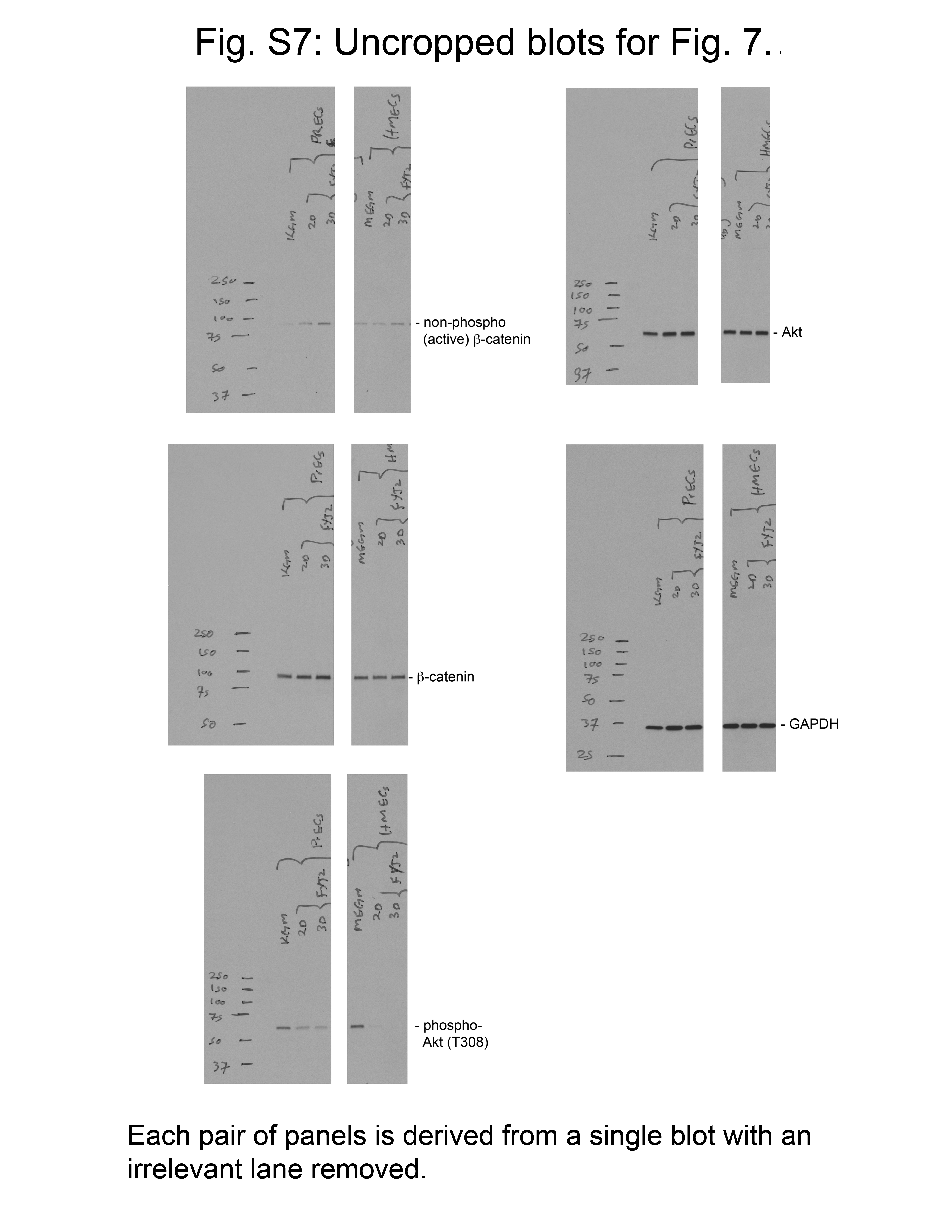

Supplement: S7 Fig — (TIF) [file pone.0180897.s007.tif]

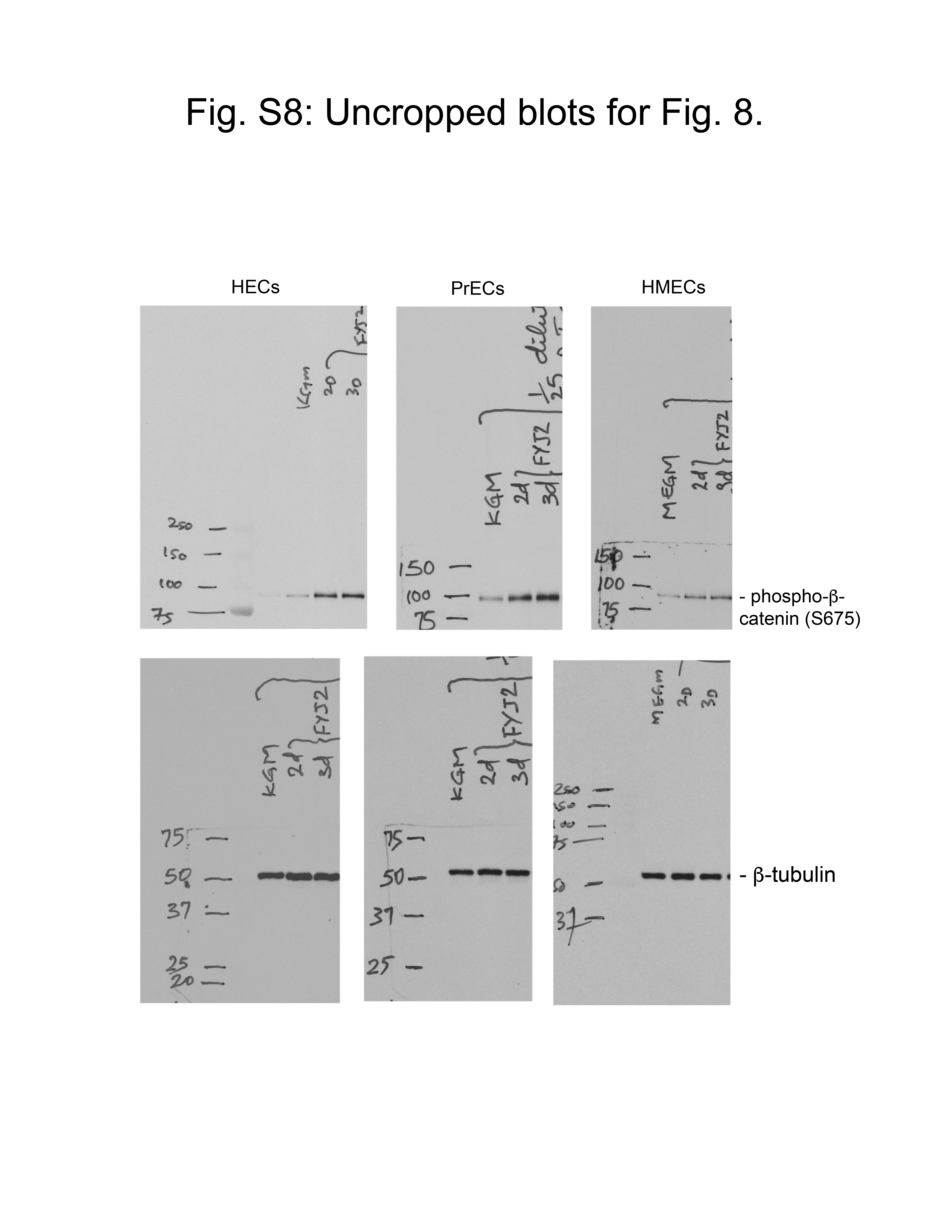

Supplement: S8 Fig — (TIF) [file pone.0180897.s008.tif]
